# Gluten and FODMAPS—Sense of a Restriction/When Is Restriction Necessary?

**DOI:** 10.3390/nu11081957

**Published:** 2019-08-20

**Authors:** Walburga Dieterich, Yurdagül Zopf

**Affiliations:** 1 Department of Medicine 1, Friedrich-Alexander-Universität Erlangen-Nürnberg, Ulmenweg 18, 91054 Erlangen, Germany; 2 Hector Center of Excellence for Nutrition, Exercise and Sports, University of Erlangen-Nürnberg, 91054 Erlangen, Germany

**Keywords:** gluten-free diet (GFD), low FODMAP diet, celiac disease, non-celiac gluten sensitivity (NCGS), irritable bowel syndrome (IBS)

## Abstract

Gluten-free diet (GFD) is enjoying increasingly popularity, although gluten-free products are considerably more expensive. GFD is absolutely necessary for patients with celiac disease, as in this case even minor amounts of gluten can lead to the destruction of the intestinal mucosa. In addition, GFD is currently the best therapy to improve clinical symptoms of patients with non-celiac gluten sensitivity (NCGS), although the diet may not be as strict as that for patients with celiac disease. Beside gluten, other wheat components such as oligosaccharides and amylase trypsin inhibitors are discussed as triggers of NCGS in this review. An overlap between gastrointestinal symptoms in NCGS and irritable bowel syndrome (IBS) is described. Patients with NCGS attribute their symptoms to the consumption of gluten, while patients with IBS rarely describe gluten as a trigger. Recently, several studies have demonstrated that the introduction of a low FODMAP (fermentable oligo-, di-, monosaccharides, and polyols) diet reduced gastrointestinal symptoms in patients with IBS and this diet is suggested as the first choice of therapy in IBS. However, a low FODMAP diet also eliminates prebiotica and may negatively influence the gut microbiota. For this reason, the diet should be liberalized after symptom improvement. There is no evidence that a GFD is healthier than the standard diet. In contrast, GFD often is accompanied by nutritional deficiencies, mainly minerals and vitamins. Therefore, GFD and low FODMAP diets are not recommended for healthy subjects. Since wheat contains fructans belonging to FODMAPs), a GFD is not only gluten-free but also has less FODMAPs. Thus, symptom improvement cannot be correctly correlated with the reduction of either one or the other.

## 1. Introduction

The market for gluten-free products has been increasing steadily and gluten-free diet (GFD) is becoming more and more popular. About 0.7–1.4% of individuals suffer from celiac disease [1] and gluten sensitivity is estimated to affect up to 6% of humans [2]. While all these patients need to follow a GFD, the vast majority of consumers of gluten-free products buy them for other reasons, e.g., a belief that the products are better for health, weight loss, or for fear of a toxic gluten effect [3,4,5]. Surveys have shown that the popularity of GFD is highest in Latin America, the Middle East, and America with 32%, 28%, and 15–21% of the population following a GFD, respectively [3]. This article summarizes who really needs a GFD and which patient groups would instead benefit from a low FODMAP diet (fermentable oligo-, di-, monosaccharides, and polyols).

## 2. Gluten-Free Diet (GFD) in Celiac Patients

Gluten-free products are an absolute must for patients with celiac disease since minor amounts of gluten can exert a destructive effect on the intestinal mucosa of celiac patients [6,7]. Gluten ingestion in genetically predisposed celiac patients, who possess the major histocompatibility surface markers DQ2 or DQ8, causes the activation of the naïve and adaptive immune system and provokes the remodeling and destruction of the intestinal mucosa. The mucosal damage is mainly found in the distal duodenum and proximal jejunum with crypt hyperplasia and villi atrophy, and finally results in a generalized malabsorption [8,9]. The spectrum of symptoms ranges from only minor clinical symptoms up to an overall malabsorption characterized by chronic severe diarrhea, weight loss, abdominal distension, and vitamin and mineral deficiency [9]. The clinical presentation of celiac disease has changed in recent years. Only one third of patients shows the typical severe symptoms characterized by malabsorption and diarrhea, whereas more and more patients suffer from non-classical gastrointestinal or extraintestinal complaints [10]. These non-classical symptoms are often responsible for long diagnostic intervals and physicians’ awareness is necessary to diagnose celiac disease without delay [11]. For a correct diagnosis, it is absolutely mandatory that patients consume a normal gluten-containing diet. To date, the diagnosis is performed by the detection of IgA class serum antibodies against transglutaminase 2 while excluding an IgA deficiency, as well as the histological confirmation of the damaged duodenal mucosa. In case of doubt, supporting information might be obtained by the detection of the genetic markers DQ2 or DQ8. However, recent studies have presented a high reliability for celiac diagnosis that is only based on the detection of increased serum antibody titers against transglutaminase 2 and endomysium and an appropriate genetic background, but without demonstration of the intestinal mucosal damage in symptomatic patients [12,13]. The future will show whether this diagnostic procedure is advisable since the renunciation of the baseline histological data will be a disadvantage, especially in cases with seronegativity or unsatisfactory histological response [14,15].

Just as manifold as the clinical appearance of celiac disease is the tolerability of daily gluten dosage, and individual patients react very differently when consuming small amounts of gluten. A safe gluten threshold has been a recent matter of discussion. A four-week gliadin provocation of celiac children, who followed a gluten-free diet for a mean of 14 months, demonstrated a significant increase in numbers of intraepithelial lymphocytes and a decrease in the ratio of villus height/crypt depth (Vh/Cd) compared to baseline already after a daily intake of 100 mg gliadin (equivalent to 200 mg gluten or 2.5 g wheat flour). The group of children that was challenged with 500 mg gliadin (corresponding to 12.5 g wheat flour) per day for four weeks showed even higher densities in intraepithelial lymphocytes, a more reduced Vh/Cd ratio, and an increased intestinal permeability [16]. Another multicenter double-blind, placebo-controlled follow-up study was performed with 49 adult patients that had been on a GFD for at least two years. The patients consumed capsules for 90 days containing 10 or 50 mg gluten or placebo. The histopathological analysis of intestinal mucosal sections demonstrated a significant decrease in the Vh/Cd ratio in patients who consumed 50 mg gluten/day and only minor changes in the 10 mg group. Based on this data, it is highly recommended to keep the daily gluten intake below 50 mg. However, one should keep in mind that the individual susceptibility to gluten is highly variable, and because some patients already showed mucosal worsening when consuming only 10 mg, limits and recommendations should be handled with care [6].

Nevertheless, some patients still suffer from gastrointestinal complaints despite adhering to a strict GFD. A recent study showed that the reduction of dietary FODMAP intake for only one month have already led to a significant improvement of quality of life and clinical symptoms in these patients. This data justify testing a low FODMAP diet in celiac patients with persistent gastrointestinal complaints while on a GFD [17]. However, there are reports that describe a positive effect of the prebiotic inulin belonging to FODMAPs. A three months supplementation with oligo-fructose enriched inulin significantly improved the vitamin D and vitamin E levels in 34 pediatric patients. In addition, an increase in *Bifidobacterium* was noticed and going along with a stimulated metabolite production thus resulting in increased fecal short chain fatty acid levels [18,19]. A dysbiosis is suggested in celiac patients even on a GFD and in this context lower numbers of bifidobacteria were detected in stool samples from celiac patients compared to healthy controls [20]. Therefore, supplementation with pre- and probiotics, e.g. *Bifidobacterium longum* might be a therapeutic option to restore a well-balanced gut microbiome and further improve health status [21,22].

## 3. Gluten-Free Diet in Non-Celiac Gluten Sensitivity (NCGS)

Patients with non-celiac gluten sensitivity (NCGS) are also recommended to adhere to a GFD. After eating gluten-containing foods, the symptoms usually appear within hours and patients complain about symptoms that resemble the clinical picture of celiac disease. In addition to gastrointestinal problems the patients often suffer from extraintestinal symptoms, such as tiredness, headache, anxiety, foggy mind, joint and muscle pain, or skin rash [23]. 

However, apart from moderately enriched numbers of intraepithelial lymphocytes in the duodenal mucosa, there is no abnormal mucosal histopathology [2,24,25]. Some reports described positivity for IgG anti-gliadin antibodies in 56.4–66% of patients, and 46% of patients possess genes for DQ2 or DQ8. However, there was no correlation of these genetic markers with IgG anti-gliadin positivity [26,27]. The lack of reliable disease specific biomarkers is the reason for the diagnosis being more difficult and prevalence data varying considerably between 0.5–6% [2,28].

Since patients already often follow a self-imposed gluten-restricted diet, they should be provoked with gluten for at least six weeks before proper a diagnosis can be performed. The diagnosis of NCGS is settled when wheat allergy and celiac disease are definitively excluded. Following a GFD for six weeks has to improve the main clinical symptoms substantially and permanently. For a correct diagnosis, a double-blind placebo-controlled challenge with 8 g of gluten per day is recommended to provoke typical NCGS symptoms. However, this approach is often difficult to perform and, especially for daily clinical practice, a single-blind procedure is suggested [23].

Although the symptoms quickly improve under GFD, gluten is not proven as the sole trigger in NCGS. In contrast, several blinded placebo-controlled studies have impugned the role of gluten in NCGS [29,30,31]. Other wheat components, such as FODMAPs, have been discussed as culprits and may be responsible for gastrointestinal symptoms, especially bloating, flatulence, and abdominal pain [29,32,33]. Recently, it has become evident that after a seven-day period of provocation most patients with self-reported NCGS have a stronger correlation between gastrointestinal symptoms and dietary fructans than with gluten [34]. In addition, amylase trypsin inhibitors (ATIs), which are naturally occurring in most cereals, may contribute to clinical symptoms in NCGS [32,35]. ATIs are able to trigger the innate immune system via the activation of monocytes by lipopolysaccharide receptor TLR4 [35,36]. In murine models, dietary ATIs worsened allergic airway inflammation [37] and enhanced allergen-induced IgE-dependent colitis and gut inflammation [38]. Since ATIs display a high resistance to heat and proteases, they are suggested as additional trigger or aggravating factor in NCGS [36].

Because it is not obvious which component of wheat is the main trigger in NCGS and since patients recover during GFD, GFD still is the current effective therapeutic option in NCGS. Patients with NCGS do not need to adhere to a GFD as strictly as patients with celiac disease. After six to eight weeks of strict elimination of gluten, NCGS patients very often tolerate minor amounts of glutens. However, the threshold values are highly variable and should be evaluated and adapted individually.

## 4. GFD in the Healthy Population

While most patients with celiac disease or NCGS would appreciate being able to consume gluten-containing food and much research is done to develop therapeutic alternatives [39,40,41], a new trend in the healthy population is to adopt a GFD. Although GFD products are considerably costlier that their gluten-containing counterparts, the sales figures of gluten-free products are increasing. Internet surveys identified that only 28% of consumers are diagnosed celiac patients and thus dependent on gluten-free foodstuff (https://www.specialtyfood.com/news/article/make-room-gluten-free/). The main reasons why healthy individuals buy gluten-free products are based on incorrect or misleading statements in popular scientific journals and chats from celebrities who suggest health benefits or weight loss during GFD. However, there are no scientific data that underline GFD as a healthier diet [42,43]. In contrast, large cohort studies found no association between gluten intake and, e.g., risk for type 2 diabetes or weight gain [44], whereas Dickey et al. observed weight gain in 81% of patients with celiac disease after a two-year period of GFD. This effect is welcome in primarily underweight celiac patients, but was also noticed in 82% of already overweight celiac patients [45]. GFD is often enriched with saturated and hydrogenated fatty acids and possesses a higher glycemic index, but a low content of alimentary fibers and 2-fold lower protein content [46,47]. A study with 93 celiac patients showed that consuming a GFD came along with a significantly higher dietary carbohydrate intake, but reduced non-starch polysaccharides [48]. A meta-analysis further revealed an increase in total cholesterol, high density lipoprotein, fasting glycaemia, and body mass index in celiac patients under GFD, which are all risk factors for cardiovascular diseases [49]. Although numerous studies showed that whole-grain consumption is associated with a reduced risk of coronary heart disease, cardiovascular, respiratory, and infectious diseases, diabetes, and overall mortality, thus underlining the beneficial effects of whole grain [50], the overall impact of GFD, normally low in fiber content, on cardiovascular diseases remains unclear [46]. An insufficient fiber intake was detected in almost all patients in a study with 50 newly diagnosed celiac patients and 55 celiac patients having consumed a GFD for more than two years. In addition, an inadequate dietary intake was also shown for thiamin, folate, vitamin A, magnesium, calcium, iron, and zinc, an effect that is mainly caused by the poor content of minerals in the raw materials usually used for gluten-free products [51,52]. This fact underlines the need for professional nutritional education for newly diagnosed patients with celiac disease to prevent nutritional deficiencies or undesired weight gain, as well as the necessity to inform healthy subjects about the risks of a self-restricted GFD.

Recently, GFD has also become very popular in athletes. A survey of 910 persons showed that 41% of Australian athletes reduce gluten intake by up to 50–100% because they believe that abdominal/gastrointestinal symptoms are caused by gluten and expect gastrointestinal well-being, an ergogenic effect, and reduced inflammation when following a GFD. The sports group with gluten restriction consisted mainly of endurance sport athletes (70%) aged between 31 and 40 years. Approximately half of the athletes who consume a GFD described an improvement in gastrointestinal symptoms alone or in combination with additional symptoms like fatigue [53]. However, a double-blind crossover study with 13 endurance cyclists who received a seven-day period of gluten-free or gluten-challenged diet revealed no differences in gastrointestinal symptoms, cytokine expression, or well-being, and no positive effect on their training performance was detected under the short-term GFD [54]. One should keep in mind that athletes, especially those with increased requirement for energy intake, e.g., during endurance exercise, might risk nutrient deficits under restricted diets like GFD; therefore, nutritional advice is also highly recommended to avoid adverse events [55].

Remarkably, quite different observations have attracted attention. People following a GFD showed significantly increased blood levels of mercury, lead, and cadmium as well as elevated concentrations of total arsenic in urine compared to subjects eating a standard diet. This effect was noticed in celiac patients as well as in healthy individuals during GFD [56]. The elevated levels for urinary arsenic and mercury in the blood were confirmed in another study [57], and even higher mercury level were reported in a study with 20 celiac patients on GFD [58]. Although the concentrations of the heavy metals did not reach the threshold limits, their permanent low-level exposure might exert detrimental effects on health [57,59]. The reason for the enriched metal concentrations in subjects following a GFD is not clear. However, it is speculated that extensive use of rice as a substitute in GFD is at least partially responsible for this effect [57].

## 5. Gluten-Free Products—Low FODMAP—Low ATI

Based on the Codex Alimentarius Standard, gluten-free goods must not exceed 20 parts per million (20 mg/kg), while <100 ppm is considered as very-low gluten. Glutens are the main storage proteins in the germ of bread wheat, rye, and barley, as well as wheat species like spelt, Durum, Emmer, Einkorn, and Kamut. Glutens are also responsible for viscoelasticity, baking behavior, and taste [60]. Gluten-free products are mainly fabricated from naturally gluten-free products, e.g., teff, millet, rice, maize, oats, or pseudocereals such as amaranth, quinoa, and buckwheat [61]. Due to the high variations of vitamin and mineral contents in pseudocereals and alternative gluten-free flours, in-depth knowledge is necessary to improve the levels of micronutrients in GFD in order to avoid nutrient deficiencies. Amaranth, quinoa, and teff are enriched in iron and magnesium [52], and the highest contents of B-vitamins are found in teff (B1), chestnut (B2), millet (B2, B3), buckwheat (B3), and amaranth flour (B6) [62].

Novel therapeutic strategies for celiac disease or gluten sensitivity aim to reduce or degrade dietary gluten. Human gastric, pancreatic, and brush-border enzymes cannot completely degrade dietary glutens because of the unusually high proportion of proline residues, and T cell stimulatory gluten peptides up to 33 amino acids in length have been found in the human gut [63]. However, numerous microorganisms are able to completely degrade gluten proteins. Based on these bacterial and fungal proteases, various drugs have already been produced and used in clinical studies with celiac patients. Although promising results have been achieved in vitro, the results of the clinical studies have so far been rather unclear and will be an issue for future study (for review, see [64,65]). Interestingly, many sourdough bacteria belonging to Streptococcaceae, Lactobacillaceae, and Bifidobacteriaceae, as well as fungi and yeasts, possess gluten-degrading enzymes. Studies have shown that the long-term fermentation of dough with selected sourdough bacteria over 24 h was highly efficient in gluten degradation, resulting in bread with an intermediate content of gluten [66,67]. While patients with celiac disease must adhere to a strict GFD, processing food to minimize gluten content might open up new possibilities for patients with NCGS.

Remarkably, naturally gluten-free cereals are not only free of gluten, but tend to have minor FODMAP contents, as shown for products made with rice, corn, quinoa, and oats. The main FODMAPs in bread are fructans and galactooligosaccharides, and the highest FODMAP levels are detected in rye and wheat, whereas low concentrations are found in spelt [68,69]. Recent studies have demonstrated that the use of sourdough organisms and a prolonged proofing time of the dough also result in a clear reduction of FODMAP concentrations [68,70]. This data might encourage the return to formerly common long-term sourdough fermentation of bread and the development of wheat-based products with low FODMAP contents, respectively, which may be better tolerated by hypersensitive subjects, e.g., patients with irritable bowel syndrome (IBS) (see below).

Furthermore, it has been shown that gluten-free substitutes such as buckwheat, oats, teff, millet, quinoa, and amaranth possess clearly reduced amylase trypsin inhibitor (ATI) bioactivity compared to wheat cultivars [36]. Interestingly, modern hexaploid wheat strains possess enriched ATI activity compared to ancient strains, such as tetraploid Emmer or diploid Einkorn [36]. In fact, mass spectroscopic analysis showed a very low or missing ATI content in eight Einkorn cultivars [71]. Because ATIs are suggested as additional triggers in NCGS, cereals without or with only a minor ATI content may be more tolerable for individuals with NCGS. Recent research has shown that sourdough fermentation has also led to reduced ATI levels. However, a double-blind seven-day pilot study with 26 patients with wheat sensitivity and irritable bowel syndrome did not found any significant differences in gastrointestinal complaints, although monomeric ATIs and FODMAPs were lower in the sourdough bread compared to yeast-fermented wheat bread. In contrast, patients reported significantly more frequent tiredness, joint symptoms, and reduced alertness when consuming the sourdough-fermented bread [72]. Thus, further studies are needed to confirm the influence of gluten, FODMAPs, ATIs, and other wheat components in NCGS.

In summary, it is important to keep in mind that a GFD made up of substitute cereals is not only free of gluten, but also has reduced FODMAP and ATI contents. In addition, since sourdough cultures are able to degrade gluten, FODMAPs, and ATIs, the time of fermentation of wheat products with appropriate sourdough cultures has a strong impact on the gluten, FODMAP, and ATI contents in the end products. Future studies are needed to prove the effectiveness of long-term sourdough baking on salubriousness and clinical symptoms in patients with NCGS.

## 6. Low FODMAP Diet for Patients with Irritable Bowel Syndrome (IBS)

The term FODMAP describes fermentable oligosaccharides, di- and monosaccharides, and polyols that are poorly absorbed in the small intestine and rapidly fermented by colonic microorganisms. The oligosaccharides include fructans (fructooligosaccharides, inulin) and galactooligosaccharides used as prebiotics. A prebiotic is defined as a “substrate that is selectively utilized by the host microorganism, conferring a health benefit” [73]. A constitutional effect of inulin, fructooligosaccharides, and galactooligosaccharides has often been described for gastrointestinal complaints, and several studies have shown a positive outcome of prebiotics on inflammatory bowel diseases or IBS, as well as on obesity, type 2 diabetes mellitus, metabolic syndrome, allergy, inflammation, and cognition, although the causality is not always clear (for review, see [73,74]). It is well known that prebiotics promote the expansion of beneficial gut organisms, e.g., *Bifidobacterium* and *Lactobacillus* species. Non-digestible prebiotics are degraded by gut bacteria and the cross-feeding of metabolites between gut microorganisms finally results in the production of short-chain fatty acids, such as butyrate, that in turn are beneficial components for host intestinal health [74,75,76]. However, the rapid fermentation of these carbohydrates by colonic bacteria also produces gases such as hydrogen, carbon dioxide, and methane that are blamed for gastrointestinal distress, mostly wind, flatulence, and abdominal pain in sensitive individuals. Dietary fructooligosaccharides, for example, increased the proportion of *Bifidobacterium* and *Lactobacillus* species, but also provoked flatulence and intestinal bloating in some subjects [77,78].

IBS is a chronic functional bowel disorder without obvious abnormalities or intestinal mucosal damages. According to Rome IV classification, patients with IBS have recurrent abdominal pain on average at least once a week for more than six months before diagnosis. The abdominal pain coincides with changes in stool form and frequency (constipation, diarrhea) or pain at defecation, and is often accompanied by flatulence and bloating [79]. Most patients with IBS suspect that the clinical symptoms are related to diet and often avoid certain foods. Therapeutic approaches in IBS have already used a FODMAP-restricted diet for a long time [80]. However, an almost complete elimination of FODMAP has been applied in several recent studies with patients suffering from IBS. The underlying idea is to avoid foods with a high FODMAP content, resulting in reduced osmotic load and bacterial gas production to minimize gastrointestinal problems in hypersensitive individuals [81].

Several studies have proven the efficacy of a low FODMAP diet with overall gastrointestinal symptom improvement in 68–86% of patients in addition to increased quality of life [81,82,83]. A multi-center single-blind study with 75 IBS patients showed that a four-week low FODMAP diet was as effective as the usual dietary advise, which recommend regular meals and minor intake of fat, insoluble fibers, caffeine or bloating foods, and that both dietary approaches significantly reduced the severity of IBS symptoms [84]. 

Although patient’s complaints significantly improved, the adherence to an eight-week low FODMAP diet was rather low in another study with 93 patients with IBS; 47% of participants stopped the diet because it was too restrictive. Remarkably, 36% of patients who finished the study showed significant weight loss [85]. Thus, appropriate nutritional advice is necessary to avoid insufficient nutrient intake and unintended weight loss. In addition, it is not known how a long-term diet will affect the intestinal microbiota. Since a low FODMAP diet also reduces the dietary amount of prebiotics, a negative effect on the intestinal microbiota cannot be ruled out. Indeed, studies have already shown a decrease in fecal *Bifidobacterium* species after a two- or four-week low FODMAP diet [24,86,87]. Additional supplementation with probiotic bacteria in combination with the low FODMAP diet in patients suffering from IBS restored fecal *Bificobacterium* species, but had no effect on clinical symptoms [86]. Nevertheless, the evidence base for a low FODMAP diet is sufficient to recommend this diet as a first-line therapy in patients with IBS [88,89]. However, the long-term stringent exclusion of FODMAPs should be avoided and the dietary restriction should be liberalized [83,90]. In this context, it is of special interest that sourdough organisms have not only the ability to degrade gluten, but also a considerable influence on the FODMAP content, and could thus open new strategies for food processing to obtain products with minor FODMAP concentrations.

**Figure 1:**
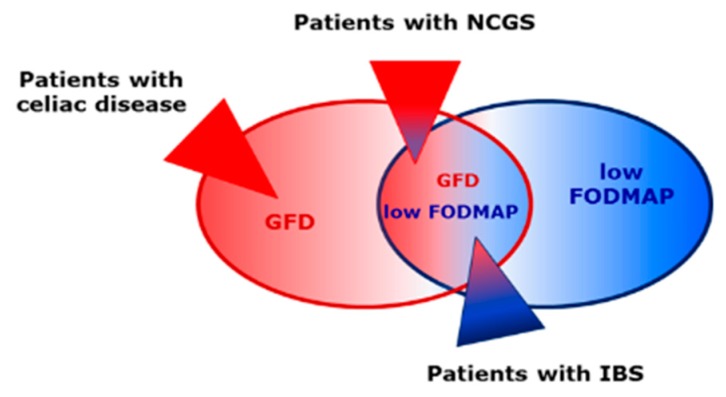
A gluten-free diet (GFD) is also reduced in fermentable oligo-, di-, monosaccharides, and polyols (FODMAPs). Patients with celiac disease must adhere to a strict GFD, while patients with non-celiac gluten sensitivity (NCGS) can tolerate minor amounts of gluten. The evidence base is sufficient to suggest a low FODMAP diet for irritable bowel syndrome (IBS). There is an overlap between NCGS and IBS symptoms. To date, no positive effects of GFD or a low FODMAP diet for healthy subjects have been proven.

An overlap between NCGS and IBS-type symptoms has been described. However, while patients with NCGS clearly correlate their symptoms with gluten consumption, patients with IBS less frequently identify gluten as trigger for gastrointestinal symptoms. Nevertheless, several studies have shown an improvement of clinical symptoms from IBS patients on GFD (for review, see [91]). As mentioned above, patients with self-reported NCGS often fail to identify gluten-containing cereals in placebo-controlled blind studies, and another study with 59 individuals revealed that dietary challenge with fructans induced more severe clinical symptoms compared to a gluten challenge [34]. Self-diagnosed NCGS patients usually eliminate gluten-containing cereals from daily diet. Since these cereals also contain FODMAPs and ATIs in addition to gluten, changing the diet also removes FODMAPs and ATIs to some extent and a reliable correlation of symptoms with gluten is no longer possible. Due to the overlapping clinical symptoms and an overlap between GFD and a low FODMAP diet, it is very likely that some patients with self-diagnosed NCGS are more likely to be IBS patients and that there is a subgroup of NCGS patients among the IBS patients [91]. See Figure 1,

## 7. Conclusions

A strict lifelong GFD is absolutely necessary for patients with celiac disease. As long as the exact trigger of NCGS is not known, GFD is currently the recommended therapy in NCGS. However, patients with NCGS do not have to adhere to a GFD as strictly as patients with celiac disease. Some studies show that patients with IBS benefit from GFD. Recent studies, however, have successfully shown the positive effect of a low FODMAP diet in IBS. That is why the low FODMAP diet is suggested as the first choice of therapy in IBS. Healthy individuals should not follow a GFD or low FODMAP diet, as malnutrition or negative long-term effects cannot be excluded.

## References

[1] Singh P., Arora A., Strand T.A., Leffler D.A., Catassi C., Green P.H., Kelly C.P., Ahuja V., Makharia G.K. (2018). Global Prevalence of Celiac Disease: Systematic Review and Meta-analysis. Clin. Gastroenterol. Hepatol..

[2] Sapone A., Bai J.C., Ciacci C., Dolinsek J., Green P.H., Hadjivassiliou M., Kaukinen K., Rostami K., Sanders D.S., Schumann M. (2012). Spectrum of gluten-related disorders: Consensus on new nomenclature and classification. BMC Med..

[3] Reilly N.R. (2016). The Gluten-Free Diet: Recognizing Fact, Fiction, and Fad. J. Pediatr..

[4] Newberry C., McKnight L., Sarav M., Pickett-Blakely O. (2017). Going Gluten Free: The History and Nutritional Implications of Today’s Most Popular Diet. Curr. Gastroenterol. Rep..

[5] Jones A.L. (2017). The Gluten-Free Diet: Fad or Necessity?. Diabetes Spectr..

[6] Catassi C., Fabiani E., Iacono G., D’Agate C., Francavilla R., Biagi F., Volta U., Accomando S., Picarelli A., De Vitis I. (2007). A prospective, double-blind, placebo-controlled trial to establish a safe gluten threshold for patients with celiac disease. Am. J. Clin. Nutr..

[7] Akobeng A.K., Thomas A.G. (2008). Systematic review: Tolerable amount of gluten for people with coeliac disease. Aliment. Pharmacol. Ther..

[8] Marsh M.N. (1995). The natural history of gluten sensitivity: Defining, refining and re-defining. QJM.

[9] Tack G.J., Verbeek W.H., Schreurs M.W., Mulder C.J. (2010). The spectrum of celiac disease: Epidemiology, clinical aspects and treatment. Nat. Rev. Gastroenterol. Hepatol..

[10] Volta U., Caio G., Stanghellini V., De Giorgio R. (2014). The changing clinical profile of celiac disease: A 15-year experience (1998-2012) in an Italian referral center. BMC Gastroenterol..

[11] Zipser R.D., Farid M., Baisch D., Patel B., Patel D. (2005). Physician awareness of celiac disease: A need for further education. J. Gen. Intern. Med..

[12] Fuchs V., Kurppa K., Huhtala H., Laurila K., Maki M., Collin P., Salmi T., Luostarinen L., Saavalainen P., Kaukinen K. (2019). Serology-based criteria for adult coeliac disease have excellent accuracy across the range of pre-test probabilities. Aliment. Pharmacol. Ther..

[13] Holmes G., Ciacci C. (2018). The serological diagnosis of coeliac disease—A step forward. Gastroenterol. Hepatol. Bed Bench.

[14] Aziz I., Peerally M.F., Barnes J.H., Kandasamy V., Whiteley J.C., Partridge D., Vergani P., Cross S.S., Green P.H., Sanders D.S. (2017). The clinical and phenotypical assessment of seronegative villous atrophy; a prospective UK centre experience evaluating 200 adult cases over a 15-year period (2000–2015). Gut.

[15] Biagi F., Vattiato C., Agazzi S., Balduzzi D., Schiepatti A., Gobbi P., Corazza G.R. (2014). A second duodenal biopsy is necessary in the follow-up of adult coeliac patients. Ann. Med..

[16] Catassi C., Rossini M., Ratsch I.M., Bearzi I., Santinelli A., Castagnani R., Pisani E., Coppa G.V., Giorgi P.L. (1993). Dose dependent effects of protracted ingestion of small amounts of gliadin in coeliac disease children: A clinical and jejunal morphometric study. Gut.

[17] Testa A., Imperatore N., Rispo A., Rea M., Tortora R., Nardone O.M., Lucci L., Accarino G., Caporaso N., Castiglione F. (2018). Beyond Irritable Bowel Syndrome: The Efficacy of the Low Fodmap Diet for Improving Symptoms in Inflammatory Bowel Diseases and Celiac Disease. Dig. Dis..

[18] Drabinska N., Jarocka-Cyrta E., Markiewicz L.H., Krupa-Kozak U. (2018). The Effect of Oligofructose-Enriched Inulin on Faecal Bacterial Counts and Microbiota-Associated Characteristics in Celiac Disease Children Following a Gluten-Free Diet: Results of a Randomized, Placebo-Controlled Trial. Nutrients.

[19] Drabinska N., Krupa-Kozak U., Abramowicz P., Jarocka-Cyrta E. (2018). Beneficial Effect of Oligofructose-Enriched Inulin on Vitamin D and E Status in Children with Celiac Disease on a Long-Term Gluten-Free Diet: A Preliminary Randomized, Placebo-Controlled Nutritional Intervention Study. Nutrients.

[20] Golfetto L., de Senna F.D., Hermes J., Beserra B.T., França Fda S., Martinello F. (2014). Lower bifidobacteria counts in adult patients with celiac disease on a gluten-free diet. Arq. Gastroenterol..

[21] De Sousa Moraes L.F., Grzeskowiak L.M., de Sales Teixeira T.F., Gouveia Peluzio Mdo C. (2014). Intestinal microbiota and probiotics in celiac disease. Clin. Microbiol. Rev..

[22] Olivares M., Castillejo G., Varea V., Sanz Y. (2014). Double-blind, randomised, placebo-controlled intervention trial to evaluate the effects of Bifidobacterium longum CECT 7347 in children with newly diagnosed coeliac disease. Br. J. Nutr..

[23] Catassi C., Elli L., Bonaz B., Bouma G., Carroccio A., Castillejo G., Cellier C., Cristofori F., de Magistris L., Dolinsek J. (2015). Diagnosis of Non-Celiac Gluten Sensitivity (NCGS): The Salerno Experts’ Criteria. Nutrients.

[24] Dieterich W., Schuppan D., Schink M., Schwappacher R., Wirtz S., Agaimy A., Neurath M.F., Zopf Y. (2019). Influence of low FODMAP and gluten-free diets on disease activity and intestinal microbiota in patients with non-celiac gluten sensitivity. Clin. Nutr..

[25] Volta U., De Giorgio R. (2012). New understanding of gluten sensitivity. Nat. Rev. Gastroenterol. Hepatol..

[26] Francavilla R., Cristofori F., Castellaneta S., Polloni C., Albano V., Dellatte S., Indrio F., Cavallo L., Catassi C. (2014). Clinical, serologic, and histologic features of gluten sensitivity in children. J. Pediatr..

[27] Volta U., Tovoli F., Cicola R., Parisi C., Fabbri A., Piscaglia M., Fiorini E., Caio G. (2012). Serological tests in gluten sensitivity (nonceliac gluten intolerance). J. Clin. Gastroenterol..

[28] DiGiacomo D.V., Tennyson C.A., Green P.H., Demmer R.T. (2013). Prevalence of gluten-free diet adherence among individuals without celiac disease in the USA: Results from the Continuous National Health and Nutrition Examination Survey 2009–2010. Scand. J. Gastroenterol..

[29] Biesiekierski J.R., Peters S.L., Newnham E.D., Rosella O., Muir J.G., Gibson P.R. (2013). No effects of gluten in patients with self-reported non-celiac gluten sensitivity after dietary reduction of fermentable, poorly absorbed, short-chain carbohydrates. Gastroenterology.

[30] Zanini B., Basche R., Ferraresi A., Ricci C., Lanzarotto F., Marullo M., Villanacci V., Hidalgo A., Lanzini A. (2015). Randomised clinical study: Gluten challenge induces symptom recurrence in only a minority of patients who meet clinical criteria for non-coeliac gluten sensitivity. Aliment. Pharmacol. Ther..

[31] Dale H.F., Hatlebakk J.G., Hovdenak N., Ystad S.O., Lied G.A. (2018). The effect of a controlled gluten challenge in a group of patients with suspected non-coeliac gluten sensitivity: A randomized, double-blind placebo-controlled challenge. Neurogastroenterol. Motil..

[32] Catassi C., Bai J.C., Bonaz B., Bouma G., Calabro A., Carroccio A., Castillejo G., Ciacci C., Cristofori F., Dolinsek J. (2013). Non-Celiac Gluten sensitivity: The new frontier of gluten related disorders. Nutrients.

[33] Van Gils T., Nijeboer P., CE I.J., Sanders D.S., Mulder C.J., Bouma G. (2016). Prevalence and Characterization of Self-Reported Gluten Sensitivity in The Netherlands. Nutrients.

[34] Skodje G.I., Sarna V.K., Minelle I.H., Rolfsen K.L., Muir J.G., Gibson P.R., Veierod M.B., Henriksen C., Lundin K.E.A. (2018). Fructan, Rather Than Gluten, Induces Symptoms in Patients With Self-Reported Non-Celiac Gluten Sensitivity. Gastroenterology.

[35] Junker Y., Zeissig S., Kim S.J., Barisani D., Wieser H., Leffler D.A., Zevallos V., Libermann T.A., Dillon S., Freitag T.L. (2012). Wheat amylase trypsin inhibitors drive intestinal inflammation via activation of toll-like receptor 4. J. Exp. Med..

[36] Zevallos V.F., Raker V., Tenzer S., Jimenez-Calvente C., Ashfaq-Khan M., Russel N., Pickert G., Schild H., Steinbrink K., Schuppan D. (2017). Nutritional Wheat Amylase-Trypsin Inhibitors Promote Intestinal Inflammation via Activation of Myeloid Cells. Gastroenterology.

[37] Zevallos V.F., Raker V.K., Maxeiner J., Scholtes P., Steinbrink K., Schuppan D. (2018). Dietary wheat amylase trypsin inhibitors exacerbate murine allergic airway inflammation. Eur. J. Nutr..

[38] Bellinghausen I., Weigmann B., Zevallos V., Maxeiner J., Reissig S., Waisman A., Schuppan D., Saloga J. (2019). Wheat amylase-trypsin inhibitors exacerbate intestinal and airway allergic immune responses in humanized mice. J. Allergy Clin. Immunol..

[39] Bakshi A., Stephen S., Borum M.L., Doman D.B. (2012). Emerging therapeutic options for celiac disease: Potential alternatives to a gluten-free diet. Gastroenterol. Hepatol. (N. Y.).

[40] Serena G., Kelly C.P., Fasano A. (2019). Nondietary Therapies for Celiac Disease. Gastroenterol. Clin. N. Am..

[41] Sollid L.M., Khosla C. (2011). Novel therapies for coeliac disease. J. Intern. Med..

[42] Gaesser G.A., Angadi S.S. (2012). Gluten-free diet: Imprudent dietary advice for the general population?. J. Acad. Nutr. Diet..

[43] Marcason W. (2011). Is there evidence to support the claim that a gluten-free diet should be used for weight loss?. J. Am. Diet. Assoc..

[44] Zong G., Lebwohl B., Hu F.B., Sampson L., Dougherty L.W., Willett W.C., Chan A.T., Sun Q. (2018). Gluten intake and risk of type 2 diabetes in three large prospective cohort studies of US men and women. Diabetologia.

[45] Dickey W., Kearney N. (2006). Overweight in celiac disease: Prevalence, clinical characteristics, and effect of a gluten-free diet. Am. J. Gastroenterol..

[46] Vici G., Belli L., Biondi M., Polzonetti V. (2016). Gluten free diet and nutrient deficiencies: A review. Clin. Nutr..

[47] Missbach B., Schwingshackl L., Billmann A., Mystek A., Hickelsberger M., Bauer G., Konig J. (2015). Gluten-free food database: The nutritional quality and cost of packaged gluten-free foods. PeerJ.

[48] Wild D., Robins G.G., Burley V.J., Howdle P.D. (2010). Evidence of high sugar intake, and low fibre and mineral intake, in the gluten-free diet. Aliment. Pharmacol. Ther..

[49] Potter M.D.E., Brienesse S.C., Walker M.M., Boyle A., Talley N.J. (2018). Effect of the gluten-free diet on cardiovascular risk factors in patients with coeliac disease: A systematic review. J. Gastroenterol. Hepatol..

[50] Aune D., Keum N., Giovannucci E., Fadnes L.T., Boffetta P., Greenwood D.C., Tonstad S., Vatten L.J., Riboli E., Norat T. (2016). Whole grain consumption and risk of cardiovascular disease, cancer, and all cause and cause specific mortality: Systematic review and dose-response meta-analysis of prospective studies. BMJ.

[51] Shepherd S.J., Gibson P.R. (2013). Nutritional inadequacies of the gluten-free diet in both recently-diagnosed and long-term patients with coeliac disease. J. Hum. Nutr. Diet..

[52] Rybicka I. (2018). The Handbook of Minerals on a Gluten-Free Diet. Nutrients.

[53] Lis D.M., Stellingwerff T., Shing C.M., Ahuja K.D., Fell J.W. (2015). Exploring the popularity, experiences, and beliefs surrounding gluten-free diets in nonceliac athletes. Int. J. Sport Nutr. Exerc. Metab..

[54] Lis D., Stellingwerff T., Kitic C.M., Ahuja K.D., Fell J. (2015). No Effects of a Short-Term Gluten-free Diet on Performance in Nonceliac Athletes. Med. Sci. Sports Exerc..

[55] Cialdella-Kam L., Kulpins D., Manore M.M. (2016). Vegetarian, Gluten-Free, and Energy Restricted Diets in Female Athletes. Sports (Basel).

[56] Raehsler S.L., Choung R.S., Marietta E.V., Murray J.A. (2018). Accumulation of Heavy Metals in People on a Gluten-Free Diet. Clin. Gastroenterol. Hepatol..

[57] Bulka C.M., Davis M.A., Karagas M.R., Ahsan H., Argos M. (2017). The Unintended Consequences of a Gluten-free Diet. Epidemiology.

[58] Elli L., Pigatto P.D., Guzzi G. (2018). Evaluation of Metals Exposure in Adults on a Gluten-Free Diet. Clin. Gastroenterol. Hepatol..

[59] Karagas M.R., Choi A.L., Oken E., Horvat M., Schoeny R., Kamai E., Cowell W., Grandjean P., Korrick S. (2012). Evidence on the human health effects of low-level methylmercury exposure. Environ. Health Perspect..

[60] Wieser H. (2007). Chemistry of gluten proteins. Food Microbiol..

[61] El Khoury D., Balfour-Ducharme S., Joye I.J. (2018). A Review on the Gluten-Free Diet: Technological and Nutritional Challenges. Nutrients.

[62] Rybicka I., Gliszczynska-Swiglo A. (2017). Gluten-Free Flours from Different Raw Materials as the Source of Vitamin B1, B2, B3 and B6. J. Nutr. Sci. Vitaminol. (Tokyo).

[63] Shan L., Molberg O., Parrot I., Hausch F., Filiz F., Gray G.M., Sollid L.M., Khosla C. (2002). Structural basis for gluten intolerance in celiac sprue. Science.

[64] Kurada S., Yadav A., Leffler D.A. (2016). Current and novel therapeutic strategies in celiac disease. Expert Rev. Clin. Pharmacol..

[65] Wungjiranirun M., Kelly C.P., Leffler D.A. (2016). Current Status of Celiac Disease Drug Development. Am. J. Gastroenterol..

[66] Rizzello C.G., Curiel J.A., Nionelli L., Vincentini O., Di Cagno R., Silano M., Gobbetti M., Coda R. (2014). Use of fungal proteases and selected sourdough lactic acid bacteria for making wheat bread with an intermediate content of gluten. Food Microbiol..

[67] Rizzello C.G., De Angelis M., Di Cagno R., Camarca A., Silano M., Losito I., De Vincenzi M., De Bari M.D., Palmisano F., Maurano F. (2007). Highly efficient gluten degradation by lactobacilli and fungal proteases during food processing: New perspectives for celiac disease. Appl. Environ. Microbiol..

[68] Muir J.G., Varney J.E., Ajamian M., Gibson P.R. (2019). Gluten-free and low-FODMAP sourdoughs for patients with coeliac disease and irritable bowel syndrome: A clinical perspective. Int. J. Food Microbiol..

[69] Biesiekierski J.R., Rosella O., Rose R., Liels K., Barrett J.S., Shepherd S.J., Gibson P.R., Muir J.G. (2011). Quantification of fructans, galacto-oligosacharides and other short-chain carbohydrates in processed grains and cereals. J. Hum. Nutr. Diet..

[70] Struyf N., Laurent J., Verspreet J., Verstrepen K.J., Courtin C.M. (2017). Saccharomyces cerevisiae and Kluyveromyces marxianus Cocultures Allow Reduction of Fermentable Oligo-, Di-, and Monosaccharides and Polyols Levels in Whole Wheat Bread. J. Agric. Food Chem..

[71] Geisslitz S., Ludwig C., Scherf K.A., Koehler P. (2018). Targeted LC-MS/MS Reveals Similar Contents of alpha-Amylase/Trypsin-Inhibitors as Putative Triggers of Nonceliac Gluten Sensitivity in All Wheat Species except Einkorn. J. Agric. Food Chem..

[72] Laatikainen R., Koskenpato J., Hongisto S.M., Loponen J., Poussa T., Huang X., Sontag-Strohm T., Salmenkari H., Korpela R. (2017). Pilot Study: Comparison of Sourdough Wheat Bread and Yeast-Fermented Wheat Bread in Individuals with Wheat Sensitivity and Irritable Bowel Syndrome. Nutrients.

[73] Gibson G.R., Hutkins R., Sanders M.E., Prescott S.L., Reimer R.A., Salminen S.J., Scott K., Stanton C., Swanson K.S., Cani P.D. (2017). Expert consensus document: The International Scientific Association for Probiotics and Prebiotics (ISAPP) consensus statement on the definition and scope of prebiotics. Nat. Rev. Gastroenterol. Hepatol..

[74] Markowiak P., Slizewska K. (2017). Effects of Probiotics, Prebiotics, and Synbiotics on Human Health. Nutrients.

[75] Gibson G.R., Probert H.M., Loo J.V., Rastall R.A., Roberfroid M.B. (2004). Dietary modulation of the human colonic microbiota: Updating the concept of prebiotics. Nutr. Res. Rev..

[76] Roberfroid M., Gibson G.R., Hoyles L., McCartney A.L., Rastall R., Rowland I., Wolvers D., Watzl B., Szajewska H., Stahl B. (2010). Prebiotic effects: Metabolic and health benefits. Br. J. Nutr..

[77] Ten Bruggencate S.J., Bovee-Oudenhoven I.M., Lettink-Wissink M.L., Katan M.B., van der Meer R. (2006). Dietary fructooligosaccharides affect intestinal barrier function in healthy men. J. Nutr..

[78] Kleessen B., Schwarz S., Boehm A., Fuhrmann H., Richter A., Henle T., Krueger M. (2007). Jerusalem artichoke and chicory inulin in bakery products affect faecal microbiota of healthy volunteers. Br. J. Nutr..

[79] Ford A.C., Lacy B.E., Talley N.J. (2017). Irritable Bowel Syndrome. N. Engl. J. Med..

[80] Mansueto P., Seidita A., D’Alcamo A., Carroccio A. (2015). Role of FODMAPs in Patients With Irritable Bowel Syndrome. Nutr. Clin. Pract..

[81] Nanayakkara W.S., Skidmore P.M., O’Brien L., Wilkinson T.J., Gearry R.B. (2016). Efficacy of the low FODMAP diet for treating irritable bowel syndrome: The evidence to date. Clin. Exp. Gastroenterol..

[82] Pedersen N., Ankersen D.V., Felding M., Wachmann H., Vegh Z., Molzen L., Burisch J., Andersen J.R., Munkholm P. (2017). Low-FODMAP diet reduces irritable bowel symptoms in patients with inflammatory bowel disease. World J. Gastroenterol..

[83] Tuck C.J., Muir J.G., Barrett J.S., Gibson P.R. (2014). Fermentable oligosaccharides, disaccharides, monosaccharides and polyols: Role in irritable bowel syndrome. Expert Rev. Gastroenterol. Hepatol..

[84] Bohn L., Storsrud S., Liljebo T., Collin L., Lindfors P., Tornblom H., Simren M. (2015). Diet low in FODMAPs reduces symptoms of irritable bowel syndrome as well as traditional dietary advice: A randomized controlled trial. Gastroenterology.

[85] Frieling T., Heise J., Krummen B., Hundorf C., Kalde S. (2019). Tolerability of FODMAP—Reduced diet in irritable bowel syndrome—Efficacy, adherence, and body weight course. Z. Gastroenterol..

[86] Staudacher H.M., Lomer M.C.E., Farquharson F.M., Louis P., Fava F., Franciosi E., Scholz M., Tuohy K.M., Lindsay J.O., Irving P.M. (2017). A Diet Low in FODMAPs Reduces Symptoms in Patients with Irritable Bowel Syndrome and a Probiotic Restores Bifidobacterium Species: A Randomized Controlled Trial. Gastroenterology.

[87] Halmos E.P., Christophersen C.T., Bird A.R., Shepherd S.J., Gibson P.R., Muir J.G. (2015). Diets that differ in their FODMAP content alter the colonic luminal microenvironment. Gut.

[88] Gibson P.R., Shepherd S.J. (2010). Evidence-based dietary management of functional gastrointestinal symptoms: The FODMAP approach. J. Gastroenterol. Hepatol..

[89] Halmos E.P., Power V.A., Shepherd S.J., Gibson P.R., Muir J.G. (2014). A diet low in FODMAPs reduces symptoms of irritable bowel syndrome. Gastroenterology.

[90] Shepherd S.J., Halmos E., Glance S. (2014). The role of FODMAPs in irritable bowel syndrome. Curr. Opin. Clin. Nutr. Metab. Care.

[91] Catassi C., Alaedini A., Bojarski C., Bonaz B., Bouma G., Carroccio A., Castillejo G., De Magistris L., Dieterich W., Di Liberto D. (2017). The Overlapping Area of Non-Celiac Gluten Sensitivity (NCGS) and Wheat-Sensitive Irritable Bowel Syndrome (IBS): An Update. Nutrients.

